# Beyond Chemotherapy, PD-1, and HER-2: Novel Targets for Gastric and Esophageal Cancer

**DOI:** 10.3390/cancers13174322

**Published:** 2021-08-27

**Authors:** Ali Zubair Siddiqui, Khaldoun Almhanna

**Affiliations:** 1 University of Mississippi Medical Center, University of Mississippi School of Medicine, Jackson, MS 39216, USA; 2 The Brown University Oncology Research Group, The Rhode Island Hospital/Lifespan Cancer Institute, Providence, RI 02903, USA; kalmhanna@lifespan.org

**Keywords:** gastric cancer, esophageal cancer, claudin, Dickkopf-related protein 1, fibroblast growth factor, matrix metalloproteinases, lenvatinib

## Abstract

**Simple Summary:**

Gastric and esophageal cancers often present at an advanced stage at diagnosis, which negatively impacts a patient’s survival. While treatment options exist, at that point, they are palliative in nature and prioritize improving survival and quality of life. The treatment paradigm for the advanced disease have been consistently changing in the right direction, but several unmet needs still exist. In response, ongoing studies are revolutionizing the approach taken to treat these cancers by focusing on targeted therapies. Current and future research aim to exploit the mechanisms responsible for enhancing cancerous growth and metastasis in order to improve patient outcomes.

**Abstract:**

Together, gastric cancer and esophageal cancer (EC) possess two of the highest incidence rates amongst all cancers. They exhibit poor prognoses in which the 5-year survival rate is dismal. In addition to cytotoxic chemotherapy, treatment efforts have been geared toward targeting human epidermal growth factor receptor 2 (HER-2), vascular endothelial growth factor (VEGF), and programmed death ligand-1 (PD-1). Although ample success has been recorded with these agents, gastric and esophageal cancer remain lethal, and further research into potential treatment alternatives is needed. In this article, we will review some of the targets at the forefront of investigation such as claudin, Dickkopf-related protein 1 (DKK-1), fibroblast growth factor receptor (FGFR), and matrix metalloproteinases (MMPs). These innovative target pathways are in the midst of clinical trials to be implemented in the treatment algorithm for this patient population. Ultimately, exploiting the oncogenic tendencies of these potential biomarkers creates an opportunity for precise treatment and improved prognosis for these cancers. Lastly, research aimed toward reversing PD-1 antibodies resistance by combining it with other novel agents or other treatment modalities is underway in order to expand existing treatment options for this patient population.

## 1. Introduction

Esophageal malignancies are common worldwide, impacting the population on a global scale. Approximately 473,000 new cases of EC were diagnosed in 2017 alone [1]. The prevalence of EC is the highest in Africa and the Middle East [2]. The aggressive nature and poor prognosis of this cancer make it a major source of cancer-related mortality worldwide [2,3]. There are two histological subtypes of EC—squamous cell carcinoma (SCC) and adenocarcinoma. Universally, SCC presents as the more dominant form of EC; however, adenocarcinoma has become the leading histological subtype in Western countries [3]. Similarly, gastric cancer has been described as one of the most common cancers worldwide [4]. In the United States, 22,000 patients are diagnosed annually and approximately half of them succumb to the disease [5]. Accessible and accurate screening is pivotal in early detection; otherwise, patients are diagnosed in later stages when treatment is palliative. Palliative chemotherapy is the main strategic approach in prolonging survival and improving quality of life [6]. Although advancements in chemotherapy have occurred, no significant improvement in survival outcomes has been demonstrated, and mortality remains high [6]. Using novel targeted therapies or combining preexisting treatments to improve management of gastric cancer and EC are at the forefront of patient care.

Targeted HER-2 therapy was one of the first success stories in utilizing targeted treatment. Trastuzumab, a monoclonal antibody targeting patients with HER-2 positive tumors led to improvement in overall survival (OS) when used in combination with chemotherapy in newly diagnosed patients [7]. Several other HER-2 targeted therapies failed to show benefits in this patient population, until recently, when fam-trastuzumab deruxtecan-nxki (Enhertu), an antibody-drug conjugate, showed promising activity in advanced GEC whose tumors expressed HER-2 [8]. These initial findings proved to be the motivation in pursuing similar targeted treatment for patients diagnosed with gastric cancer or EC.

In the last decade, immunotherapy using immune-checkpoint inhibitors (ICI) has revolutionized cancer treatment. Led by its first approval in melanomas, ICIs have been approved for use in both metastatic and adjuvant settings for several malignancies. The US Food and Drug Administration (FDA) recently approved ICIs such as Keytruda (pembrolizumab) [9] and Opdivo (nivolumab) [10], in combination with chemotherapy, for the initial treatment of patients with advanced or metastatic gastric, esophageal, or gastroesophageal junction (GEJ) cancer. Additionally, Nivolumab was approved by the FDA for patients with completely resected esophageal or GEJ cancer that had residual pathologic disease and received neoadjuvant chemoradiotherapy [11]. ICIs are currently being tested far beyond advanced disease, and the results from several ongoing studies in the neoadjuvant and adjuvant settings will be awaited.

**Figure 1:**
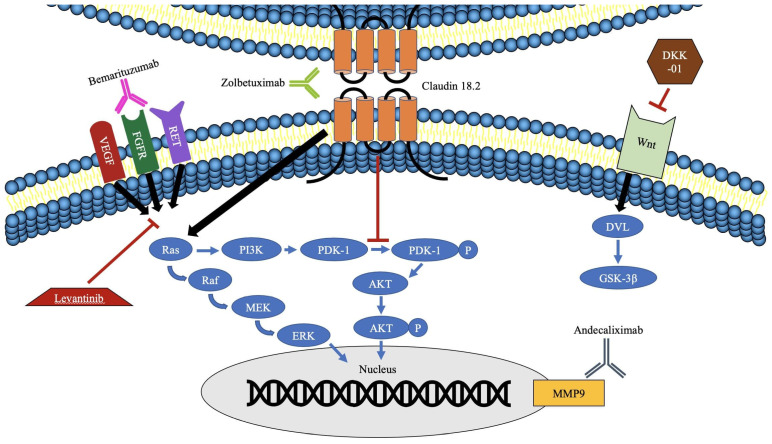
Novel antibodies as anticancer agents. Akt: protein kinase B, DKK-01: Dickkopf-related protein 1, DVL: disheveled protein, ERK: extracellular receptor kinase, FGFR: fibroblast growth factor receptor, GSK-3β: glycogen synthase kinase 3 beta, MEK: mitogen-activated protein, MMP9: matrix metalloproteinase 9, PDK-1: phosphoinositide-dependent kinase-1, PI3K: phosphoinositide 3-kinase, Raf: rapidly accelerated fibrosarcoma, VEGF: vascular endothelial growth factor, Wnt: portmanteau of int and wg.

The aim of this article is to discuss other novel agents currently being examined in the treatment of gastric cancer and EC. Most agents discussed here have shown promising results in early stage trials and are currently being tested alone or against the standard of care in randomized trials (Table 1). The exact role of these agents is yet to be determined as the landscape of treatment is changing, especially in the front line. We will review claudin, Dickkopf-related protein 1 (DKK-1), fibroblast growth factor receptor (FGFR), and matrix metalloproteinases (MMPs) (Figure 1). In addition, we will discuss PD-1 antibody resistance, a known phenomenon, and the intensive efforts to oppose the resistance or improve its activity by combining it with other agents or other treatment modalities.

## 2. Targeting Claudin 18.2 with Zolbetuximab (IMAB362)

Claudins are tetraspan transmembrane proteins of tight junctions associated with several functions in maintaining the integrity of cell–cell adhesion [12]. Claudin 18.2 (CLDN18.2) is located specifically in gastric mucosal cells and, under normal conditions, its epitopes are typically isolated from the external environment [13]. This prevents any therapeutic strategy involving intravenous antibodies. However, gastric mucosal cell malignancy results in CLDN18.2 epitope exposure and enables it to become a target unique to cancer cells [13]. Zolbetuximab is a chimeric monoclonal antibody that targets CLDN18.2 epitopes and initiates cell death by antibody-dependent cellular cytotoxicity and complement-dependent cytotoxicity [17]. In a phase I trial, Zolbetuximab showed single-agent efficacy and manageable toxicity in patients with heavily pretreated advanced CLDN18.2-positive gastric/gastroesophageal junction (G/GEJ) cancer [17]. Furthermore, in a phase II trial, Zolbetuximab monotherapy exhibited antitumor activity in patients sharing the same cancer parameters [18]. Based on preclinical trials demonstrating Zolbetuximab’s capacity to cooperate with other cytotoxic agents, a phase II trial randomizing patients with advanced G/GEJ and esophageal adenocarcinoma and CLDN18.2 expression of at least 40% to receive epirubicin + oxaliplatin + capecitabine (EOX) vs, zolbetuximab + EOX every 3 weeks was conducted [19]. The trials showed a significant improvement in progression-free survival (PFS) and OS when a patient received Zolbetuximab + EOX, compared to EOX alone. The improvement observed in PFS was exclusively noted in patients expressing upwards of 70% CLDN18.2 in malignant gastric mucosal cells. Adverse events (AEs) were limited to grade 1 and 2 experiences of nausea, vomiting, neutropenia, and anemia. No increase in grade 3 AEs was observed [19]. Currently, a phase III trial (SPOTLIGHT) for patients with moderate-to-strong CLDN18.2 expression is ongoing, and the results will be awaited.

## 3. Targeting Matrix Metalloproteinases (MMPs)

Matrix metalloproteinases (MMPs) are a group of zinc-dependent proteases responsible for degrading and remodeling the extracellular matrix (ECM) and basement membrane under normal physiological conditions [20]. They are secreted as inactive proproteins, which are activated when cleaved by extracellular proteinases. MMP9 is a specific member of this group and its expression by tumor epithelia regulates inflammation, ECM remodeling, and neovascularization [14]. MMP9 expression has been associated with loss-of-tumor suppression activity, as well as with gain-of-oncogenic activity [14,20]. The extensive role MMP9 plays in tumor progression allows it to be an excellent target for treatment in gastric adenocarcinoma. Andecaliximab (ADX) is a recombinant chimeric immunoglobulin G4 monoclonal antibody that is highly specific to MMP9. In a phase I/Ib study, the combination of ADX (800 mg every 2 weeks) and fluorouracil, leucovorin, and oxaliplatin (mFOLFOX6) showed improved activities in patients with G/GEJ adenocarcinoma with a PFS of 9.9 months in the front-line patients and response rates of 48% [21]. These initial findings led to a phase III study examining the efficacy of the FOLFOX/ADX combination vs. mFOLFOX6/Placebo (PBO) in patients with HER2- negative gastric or GEJ adenocarcinoma [15]. Unfortunately, the trial showed no statistically significant difference in OS when ADX was added to mFOLFOX6 (the median OS was 12.5 versus 11.8 months in the ADX and PBO groups, respectively. The median PFS was 7.5 versus 7.1 months in the ADX and PBO groups, respectively).

## 4. Targeting Fibroblast Growth Factor Receptor FGFR

The fibroblast growth factor receptor (FGFR) is a receptor tyrosine kinase responsible for angiogenesis, embryogenesis, tissue homeostasis, and wound repair. Furthermore, it regulates proliferation, differentiation, apoptosis, and migration [22]. Mutations in this receptor are correlated with oncogenic activity, suggesting its potential use as a target in therapy [22]. Bemarituzumab is a first-in-class, humanized IgG1 monoclonal antibody that selectively binds to FGFR2b. Bemarituzumab was recently evaluated in a global, randomized, double-blind, placebo-controlled phase II FIGHT trial of patients with newly diagnosed metastatic HER-2 negative advanced gastric or gastroesophageal cancer. To be eligible for the trial, the tumor should express FGFR2b or have *FGFR2* genetic amplification [23]. A total of 155 patients were treated with modified FOLFOX6 plus/minus bemarituzumab 15 mg/kg or placebo every 2 weeks with one additional bemarituzumab dose of 7.5 mg/kg on day 8. The primary endpoint was PFS. The addition of bemarituzumab led to improvement in PFS from 7.4 months to 9.5 months (hazard ratio [HR] = 0.68, *p* = 0.07). The secondary endpoint of OS was also met, with the median not being reached in the bemarituzumab arm, compared to 12.9 months in the control arm (HR = 0.58, *p* = 0.03). Response rates increased from 40% to 53%, with a median duration of response of 7.1 months with placebo vs. 12.2 months with bemarituzumab. Subgroup analysis showed the response correlated with immunohistochemistry (IHC) staining of FGFR2b. Patients who received bemarituzumab had a higher rate of toxicity, specifically ocular toxicity. These results support a prospective randomized phase III study in gastric/gastroesophageal adenocarcinoma [23].

## 5. Targeting DKK-01

The Wnt signaling pathway has major implications in cell fate decisions, proliferation, and migration pathways [24]. The DKK-01 protein is a potent antagonist in the Wnt signaling pathway, and its expression is directly correlated with increased tumor growth and angiogenesis [24,25]. DKN-01 is an effective antibody responsible for negating the activity of DKK-01 protein, which is a modulator of Wnt/beta-catenin and CKAP4/PI3K/AKT signaling pathways and is frequently implicated in tumorigenesis [26]. In a phase I/II trial consisting of previously treated patients with advanced esophagogastric cancer, DKN-01 monotherapy and its combination with paclitaxel or pembrolizumab were evaluated [27]. The combination of DKN-01 and pembrolizumab resulted in favorable outcomes for patients with gastric/GEJ tumors that had high DKK1 expression and for those who had not received previous treatment with a PD-1 or PD-L1 inhibitor. A median PFS over 22 weeks and median OS of 32 weeks were observed. The objective response rate (ORR) elicited with the combination treatment was 50% in these patients, and the disease control rate (DCR) was 80%. Moreover, in patients with low DKK1 expression, the median PFS with the combination treatment was approximately 6 weeks, the median OS was over 17 weeks, and the DCR was 20%. High expression of DKK1 was linked with longer PFS independent of PD-L1 combined positive score (CPS) levels [27]. Currently, an ongoing phase IIa DisTinGuish study (NCT04363801) is examining DKN-01 in combination with tislelizumab (BGB-A317) with or without chemotherapy as a first- or second-line treatment in adult patients with inoperable, locally advanced gastric/GEJ adenocarcinoma.

## 6. Manipulating PD-1 Antibodies

PD-1 sits on the surface of T cells and its interaction with programmed cell death ligand-1 (PD-L1) enables the cell to bypass immune checkpoints and avoid immune recognition [28]. This prevents T cell proliferation and its effector functions such as tumor cell-killing. Under oncogenic circumstances, tumor cells increase the expression of PD-1 to evade this inhibitory checkpoint and enhance cancerous growth [29]. Creating targeted antibodies for the PD-1 receptor prevents a cancer cell from avoiding an immune response, but these antibodies are faced with resistance. Revising PD-1 antibodies resistance has been the subject of several preclinical and clinical trials with limited success. Another approach would be to further enhance immunological and clinical responses using combinatorial approaches with chemotherapy and radiation, cytokines, and co-stimulatory agents [28]. This is currently the subject of several ongoing trials in GEC, and the results will be awaited.

## 7. Combination with CTLA-4 Antibodies

Cytotoxic T-lymphocyte-associated protein 4 (CTLA-4) is an inhibitory checkpoint activated in T cells that downregulate immune responses [30]. Considering PD-1 shares a similar role, these two checkpoints represent an opportunity for targeted therapy in an antitumor response. A combination of a CTLA-4 blocker (ipilimumab) and a PD-1 blocker (nivolumab) have demonstrated clinical benefits in patients diagnosed with metastatic melanoma, advanced renal cell carcinoma, or metastatic colorectal cancer [30]. The synergistic effect of merging these two treatment options has been tested with several challenges and specific toxicities. Dosing of the combination has been hard to tolerate and has manifested into patients experiencing significant side effects [31]. The initial efforts in combining these treatments occurred during a phase I/II checkmate-032 study. Patient criteria included individuals diagnosed with advanced or metastatic refractory gastric, esophageal, or gastroesophageal junction cancer. The primary endpoint for this study was objective response rate (ORR) for patients receiving either 1 mg/kg nivolumab plus 3 mg/kg ipilimumab, 3 mg/kg nivolumab plus 1 mg/kg ipilimumab, or 3 mg/kg nivolumab. The ORR for these patient groups were 24%, 8%, and 12%, respectively. The 12-month progression-free survival rates were 17%, 10%, and 8%, respectively. Meanwhile, overall survival of 12 months was 35%, 24%, and 39%, respectively. This study concluded that nivolumab and the combination of nivolumab plus ipilimumab have clinical significance in antitumor activity [31].

The combination of nivolumab and ipilimumab was also evaluated in checkmate-649 [10], which was a phase III trial assessing nivolumab plus chemotherapy or ipilimumab versus chemotherapy alone as a first-line treatment in patients with non-HER-2-positive advanced gastric, gastroesophageal junction, or esophageal cancer—all with adenocarcinoma histology. In the nivolumab/ipilimumab arm, patients received treatment with nivolumab 1 mg/kg every 2 weeks and ipilimumab 3 mg/kg. This arm of the study was terminated because of toxicity, and the efficacy results are yet to be presented [10].

The same combination was also evaluated to improve clinical outcomes in metastatic SCC of the esophagus in a phase III checkmate-648 [16] study against chemotherapy alone in patients with advanced esophageal squamous cell carcinoma (ESCC) as part of a three-arm study. Patients received treatment with nivolumab 3 mg/kg every 2 weeks and ipilimumab 1 mg/kg every 6 weeks up to 24 months or until disease progression or unacceptable toxicity. Progression-free survival with nivolumab plus ipilimumab vs. chemotherapy in patients with tumor cell PD-L1 ≥ 1% did not meet the prespecified boundary for significance. The objective response rate was 35% in the nivolumab/ipilimumab, compared to 20% in the chemotherapy-only arm in patients with tumor cell PD-L1 ≥ 1%. These promising results could provide the benefit of having the first dual immunotherapy combination to demonstrate benefit in ESCC, which could be a preferable non-chemotherapy option for patients.

## 8. Combination with Lenvatinib

Lenvatinib targets and inhibits vascular endothelial growth factor (VEGF) and fibroblast growth factor (FGF) tyrosine kinase receptors. Preclinical trials demonstrated lenvatinib’s ability to function as an antitumor treatment against varying solid tumor types [32]. It is believed that the tumor microenvironment is a critical point at which resistance to checkpoint inhibition occurs [32]. Evidence suggests intervening treatment can modify this environment, and that the tumor microenvironment can predict the response to a checkpoint blockade [32]. When lenvatinib is administered with a PD-1 blockade, its activity is strengthened in the tumor microenvironment [33]. Specifically, lenvatinib decreases monocyte and macrophage numbers while increasing cytotoxic T cell populations [33]. A phase II study reported a response rate of 69% when lenvatinib was combined with a pembrolizumab as first- or second-line treatment in gastric cancer. This surpassed the efficacy of either treatment when used alone [34]. Responses were observed in both CPS > 1 (16/19–84%) and CPS < 1 (4/10–40%) groups. This suggests a profound effect of lenvatinib on the microenvironment leading to increased pembrolizumab activity. In the gastric/esophageal arm of the LEAP study, the combination showed more modest benefits with a response rate of 39% and PFS of 7.4 months [35]. This combination will be tested further in the future.

## 9. Incorporating PD-1 Antibodies with Chemotherapy and Radiation

The integration of immunotherapy, in combination with radiation therapy into the treatment of advanced locoregional disease, has been evaluated in several diseases with varying degrees of benefits. In this setting, immunotherapies might have their greatest potential to affect patient survival and achieve curative outcomes. It has been hypothesized that the interaction of radiation with the immune system and the potential to augment antitumor immunity might be beneficial. Radiation has the potential to increase the susceptibility of tumor cells to immune-mediated killing [36] by upregulating the negative feedback. Based on this fact, a multicenter, randomized phase II study of neoadjuvant pembrolizumab plus chemotherapy and chemoradiotherapy in esophageal adenocarcinoma was recently presented at ASCO 2021 meeting. Patients with at least cT3 disease or N+ disease who are eligible for curative surgery received concurrent chemotherapy and radiation with weekly carboplatin and paclitaxel in addition to pembrolizumab every 3 weeks after initial induction treatment with chemotherapy +/- pembrolizumab. Following resection, patients received pembrolizumab for one year. The primary endpoint was the rate of major pathologic response (MPR), defined as a complete pathologic response or near-complete response (<10% residual cancer). A total of 40 patients were enrolled; notable toxicity included grade 3–4 pneumonitis (13%), anastomotic leak (13%), infection (35%). In 31 evaluable patients to date, the MPR rate was 50.0% (95% CI, 33–67%). One-year disease-free survival was 100% for patients with MPR vs. 32% without MPR (*p* = 0.002). The study concluded that the addition of pembrolizumab to preoperative CRT is safe and associated with a significantly higher MPR rate, compared to historical data [37].

Currently, an ongoing phase III study is utilizing nivolumab, ipilimumab, and radiation therapy for patients with esophageal and gastroesophageal junction adenocarcinoma who are candidates to undergo surgery (NCT03604991). Recruitment for the trial is ongoing and intends on demonstrating both the efficacy of this treatment and the clinical difference in administering it in a neoadjuvant or adjuvant setting. The primary outcomes in the study focus on observing a complete pathologic response and disease-free survival. Secondary outcomes consist of recording the incidence of adverse events and overall survival. Results will be awaited.

## 10. Conclusions

EC is the sixth leading cause of cancer-related deaths worldwide, and gastric cancer is currently third [38]. In the past, the main treatment for the advanced disease was through palliative chemotherapy [6]; however, recent innovations in targeted therapy have demonstrated promising results in improving the prognosis of both. For the last decade, trastuzumab has been the only approved front-line targeted therapy for metastatic HER-2 positive disease, until recently, when ENHERTU was approved for second-line treatment [8]. Ramucirumab, a VEGF antibody, was approved as a single agent or in combination with paclitaxel as a second-line treatment and failed to improve outcome in the front line [39]. Then, came the ICI era during which progress was made in several malignancies including esophageal and gastric cancer. Currently, ICIs are approved in combination with chemotherapy in front-line treatment. Questions yet to be answered regarding ICIs include the mechanisms of resistance to these agents, patient selection, the order in which these agents will be offered given the ongoing changes in the treatment paradigm, and whether a combination approach would provide better patient outcomes.

Parallel efforts in trying to investigate other agents are ongoing in both clinical and preclinical settings. In this article, we summarized the novel agents that are currently being tested in the front- and second-line treatment for EC and gastric cancer. Additionally, we briefly reviewed some of the efforts of combining ICIs with other agents or treatment modalities to improve outcomes. The majority of the new agents are being evaluated in special patient populations to help guide the best approach for the integration of these novel targeted therapies into clinical settings.

As more data become accessible, the place of these agents in the treatment paradigm is expected to evolve. After success in the second and third lines, some of these agents might advance to earlier lines of treatment once their activity is confirmed. For example, certain combinations (i.e., nivolumab plus ipilimumab) may be a reasonable first-line treatment option for patients who are unsuitable for standard-of-care platinum-based therapy [31]. Moreover, the use of molecular testing as an enrichment strategy to treat selected patients with claudin antibodies in the front line instead of PD-1 antibody is currently being tested (NCT03504397). The best approach in patients harboring two mutations or expressing two antigens (HER-2 and DKK-1 for example) is not currently known. Those patients might be candidates for combination therapy in the 1L setting if data were available. However, the safety and feasibility of these combinations will need to be studied, and adverse events should be monitored closely.

Lastly, the role of these agents in the adjuvant or neoadjuvant setting is yet to be established. Targeting HER-2 has failed in the neoadjuvant setting in EC [40], but other agents may improve the outcome, leaving the opportunity for them to become an important treatment option in earlier-stage disease. Exploring the combination of these agents with radiation therapy in early stage disease is, and will be, the subject of several future trials.

After decades of research into targeted therapies for gastric and esophageal cancers, several advances have been achieved in the last several years. Initial success in these trials has prompted further investigations. Much research remains to be conducted in identifying a regimen fit to handle the high morbidities associated with both gastric cancer and EC. Incorporating these new frontline treatments are the impetus to improving survival and quality of life for patients diagnosed with these cancers. While the future looks promising, more efforts are still needed to optimize outcomes.

## Figures and Tables

**Table 1:** Summary of the novel agents being tested in gastric and gastroesophageal junction. MMP: metalloproteinases, FGFR: fibroblast growth factor receptor. DKK-01: Dickkopf-related protein 1. OS. Overall survival. PFS: progression-free survival. M: months.

Target	Agent	Mechanism of Action	Phase Study	Line of Therapy	Approved Yes/No	Outcome
Claudin 18.2 [12]	zolbetuximab	chimeric monoclonal antibody	SPOTLIGHT Phase III	First	No	Still accruing
MMP [13]	Andecaliximab	recombinant chimeric G4 Antibody	GAMMA-1 Phase III	First	No	OS 12.5 vs. 11.8
FGFR [14]	Bemarituzumab	IgG1 monoclonal Antibody	FIGHT Phase II	First	No	OS. Not reached vs. 12.9 m
DKK-01 [15]	DKN-01	monoclonal Antibody	Phase II	Second	No	Still accruing
Multiple Kinase [16]	Lenvatinib	Multikinase inhibitor	Leap Phase II	Second and beyond	No	PFS 7.4 m

## References

[1] (2020). The global, regional, and national burden of oesophageal cancer and its attributable risk factors in 195 countries and terri-tories, 1990–2017: A systematic analysis for the Global Burden of Disease Study 2017. Lancet. Gastroenterol. Hepatol..

[2] Domper Arnal M.J., Ferrández Arenas Á., Lanas Arbeloa Á. (2015). Esophageal cancer: Risk factors, screening and endoscopic treat-ment in Western and Eastern countries. World J. Gastroenterol..

[3] Abbas G., Krasna M. (2017). Overview of esophageal cancer. Ann. Cardiothorac. Surg..

[4] Jemal A., Bray F., Center M., Ferlay J., Ward E., Forman D. (2011). Global cancer statistics. CA Cancer J. Clin..

[5] Siegel R.L., Miller M.D., Fuchs H.E., Jemal A. (2021). Cancer Statistics, 2021. CA Cancer J. Clin..

[6] Ekheden I., Ebrahim F., Ólafsdóttir H., Rasschou P., Wettermark B., Henriksson R., Ye W. (2020). Survival of esophageal and gastric cancer patients with adjuvant and palliative chemotherapy-a retrospective analysis of a register-based patient cohort. Eur. J. Clin. Pharma. Col..

[7] Bang Y.J., Cutsem E.V., Feyereislova A., Chung H.C., Shen L., Sawaki A., Lordick F., Ohtsu A., Omuro Y., Satoh T. (2010). Trastuzumab in combination with chemotherapy versus chemotherapy alone for treatment of HER2-positive advanced gastric or gas-tro-oesophageal junction cancer (ToGA): A phase 3, open-label, randomised controlled trial. Lancet.

[8] Shitara K., Bang Y.-J., Iwasa S., Sugimoto N., Ryu M.-H., Sakai D., Chung H.-C., Kawakami H., Yabusaki H., Lee J. (2020). Trastuzumab Deruxtecan in Previously Treated HER2-Positive Gastric Cancer. N. Engl. J. Med..

[9] Li Z., Sun Y., Ye F., Ma D., Yin X., Zhuang W., Yuan X., Qin S., Zhang Y., Gu K. (2021). First-line pembrolizumab plus chem-otherapy versus chemotherapy in patients with advanced esophageal cancer: Chinese subgroup analysis of KEYNOTE-590. J. Clin. Oncol..

[10] Moehler M.H., Shitara K., Garrido M., Salman P., Shen L., Wyrwicz L., Yamaguchi K., Skoczylas T., Bragagnoli A.S.C., Liu T. (2021). First-line (1L) nivolumab (NIVO) plus chemotherapy (chemo) versus chemo in advanced gastric cancer/gastroesophageal junction cancer/esophageal adenocarcinoma (GC/GEJC/EAC): Expanded efficacy and safety data from CheckMate 649. J. Clin. Oncol..

[11] Kelly R.J., Ajani J.A., Kuzdzal J., Zander T., Van Cutsem E., Piessen G., Mendez G., Feliciano J., Motoyama S., Lièvre A. (2021). Adjuvant Nivolumab in Resected Esophageal or Gastroesophageal Junction Cancer. N. Engl. J. Med..

[12] Chen Y.-H., Ding L., Lu Z., Lu Q. (2013). The claudin family of proteins in human malignancy: A clinical perspective. Cancer Manag. Res..

[13] Farina A.R., Mackay A.R. (2014). Gelatinase B/MMP-9 in Tumour Pathogenesis and Progression. Cancers.

[14] Chae Y.K., Ranganath K., Hammerman P.S., Vaklavas C., Mohindra N., Kalyan A., Matsangou M., Costa R., Carneiro B., Villaflor V.M. (2017). Inhibition of the fibroblast growth factor receptor (FGFR) pathway: The current landscape and barriers to clin-ical application. Oncotarget.

[15] Park H., Jung H.Y., Choi H.-J., Kim D.Y., Yoo J.-Y., Yun C.-O., Min J.-K., Kim Y.-M., Kwon Y.-G. (2014). Distinct roles of DKK1 and DKK2 in tumor angiogenesis. Angiogenesis.

[16] Kimura T., Kato Y., Ozawa Y., Kodama K., Ito J., Ichikawa K., Yamada K., Hori Y., Tabata K., Takase K. (2018). Immuno-modulatory activity of lenvatinib contributes to antitumor activity in the Hepa1-6 hepatocellular carcinoma model. Cancer Sci..

[17] Jia X., Lu M., Rui C., Xiao Y. (2019). Consensus-Expressed CXCL8 and MMP9 Identified by Meta-Analyzed Perineural Invasion Gene Signature in Gastric Cancer Microarray Data. Front. Genet..

[18] Shah M.A., Starodub A., Sharma S., Berlin J., Patel M., Wainberg Z.A., Chaves J., Gordon M., Windsor K., Brachmann C.B. (2018). Andecaliximab/GS-5745 Alone and Combined with mFOLFOX6 in Advanced Gastric and Gastroesophageal Junction Ade-nocarcinoma: Results from a Phase I Study. Clin. Cancer Res..

[19] Shah M.A., Bodoky G., Starodub A., Cunningham D., Yip D., Wainberg Z.A., Bendell J., Thai D., He J., Bhargava P. (2021). Phase III Study to Evaluate Efficacy and Safety of Andecaliximab With mFOLFOX6 as First-Line Treatment in Patients With Ad-vanced Gastric or GEJ Adenocarcinoma (GAMMA-1). J. Clin. Oncol..

[20] Özlem T., Koslowski M., Helftenbein G., Castle J., Rohde C., Dhaene K., Seitz G., Sahin U. (2011). Claudin-18 gene structure, regulation, and expression is evolutionary conserved in mammals. Gene.

[21] Wainberg Z.A., Enzinger P.C., Kang Y.-K., Yamaguchi K., Qin S., Lee K.-W., Oh S.C., Li J., Turk H.M., Teixeira A.C. (2021). Randomized double-blind placebo-controlled phase 2 study of bemarituzumab combined with modified FOLFOX6 (mFOLFOX6) in first-line (1L) treatment of advanced gastric/gastroesophageal junction adenocarcinoma (FIGHT). J. Clin. Oncol..

[22] Sahin U., Schuler M., Richly H., Bauer S., Krilova A., Dechow T., Jerling M., Utsch M., Rohde C., Dhaene K. (2018). A phase I dose-escalation study of IMAB362 (Zolbetuximab) in patients with advanced gastric and gastro-oesophageal junction cancer. Eur. J. Cancer.

[23] Kagey M.H., He X. (2017). Rationale for targeting the Wnt signalling modulator Dickkopf-1 for oncology. Br. J. Pharmacol..

[24] Türeci O., Sahin U., Schulze-Bergkamen H., Zvirbule Z., Lordick F., Koeberle D., Thuss-Patience P., Ettrich T., Arnold D., Bassermann F. (2019). A multicentre, phase IIa study of zolbetuximab as a single agent in patients with recurrent or refractory ad-vanced adenocarcinoma of the stomach or lower oesophagus: The MONO study. Ann. Oncol..

[25] Wall J.A., Klempner S.J., Arend R.C. (2020). The anti-DKK1 antibody DKN-01 as an immunomodulatory combination partner for the treatment of cancer. Expert. Opin. Investig. Drugs.

[26] Klempner S.J., Bendell J.C., Villaflor V.M., Tenner L.L., Stein S., Naik G.S., Sirard C.A., Kagey M., Chaney M.F., Strickler J.H. (2020). DKN-01 in combination with pembrolizumab in patients with advanced gastroesophageal adenocarcinoma (GEA): Tumoral DKK1 expression as a predictor of response and survival. J. Clin. Oncol..

[27] Sato H., Okonogi N., Nakano T. (2020). Rationale of combination of anti-PD-1/PD-L1 antibody therapy and radiotherapy for cancer treatment. Int. J. Clin. Oncol..

[28] Zhang T., Song X., Xu L., Ma J., Zhang Y., Gong W., Zhang Y., Zhou X., Wang Z., Wang Y. (2018). The binding of an anti-PD-1 antibody to FcγRΙ has a profound impact on its biological functions. Cancer Immunol. Immunother..

[29] Rotte A. (2019). Combination of CTLA-4 and PD-1 blockers for treatment of cancer. J. Exp. Clin. Cancer Res..

[30] Janjigian Y.Y., Bendell J., Calvo E., Kim J.W., Ascierto P.A., Sharma P., Ott P.A., Peltola K., Jaeger D., Evans J. (2018). CheckMate-032 Study: Efficacy and Safety of Nivolumab and Nivolumab Plus Ipilimumab in Patients with Metastatic Esophagogastric Cancer. J. Clin. Oncol..

[31] Ajani J.A., Kato K., Doki Y., Chau I., Xynos I., Balogh A., Kitagawa Y. (2018). CheckMate 648: A randomized phase 3 study of nivolumab plus ipilimumab or nivolumab combined with fluorouracil plus cisplatin versus fluorouracil plus cisplatin in patients with un-resectable advanced, recurrent, or metastatic previously untreated esophageal squamous cell carcinoma. J. Clin. Oncol..

[32] Sahin U., Türeci Ö., Manikhas G., Lordick F., Rusyn A., Vynnychenko I., Dudov A., Bazin I., Bondarenko I., Melichar B. (2021). FAST: A randomised phase II study of zolbetuximab (IMAB362) plus EOX versus EOX alone for first-line treatment of advanced CLDN18.2-positive gastric and gastro-oesophageal adenocarcinoma. Ann. Oncol..

[33] Kato Y., Tabata K., Kimura T., Yachie-Kinoshita A., Ozawa Y., Yamada K., Ito J., Tachino S., Hori Y., Matsuki M. (2019). Lenvatinib plus anti-PD-1 antibody combination treatment activates CD8+ T cells through reduction of tumor-associated macrophage and activation of the interferon pathway. PLoS ONE.

[34] Kawazoe A., Fukuoka S., Nakamura Y., Kuboki Y., Wakabayashi M., Nomura S., Mikamoto Y., Shima H., Fujishiro N., Higuchi T. (2020). Lenvatinib plus pembrolizumab in patients with advanced gastric cancer in the first-line or second-line setting (EPOC1706): An open-label, single-arm, phase 2 trial. Lancet Oncol..

[35] Taylor M.H., Schmidt E.V., Dutcus C., Pinheiro E.M., Funahashi Y., Lubiniecki G., Rasco D. (2021). The LEAP program: Lenvatinib plus pembrolizumab for the treatment of advanced solid tumors. Futur. Oncol..

[36] Jagodinsky J.C., Harari P.M., Morris Z.S. (2020). The Promise of Combining Radiation Therapy with Immunotherapy. Int. J. Radiat. Oncol..

[37] Shah M.A., Almhanna K., Iqbal S., Thakkar P., Schneider B.J., Yantiss R., Wu Y., Futamura E., Port J.L., Spinelli C. (2021). Multicenter, randomized phase II study of neoadjuvant pembrolizumab plus chemotherapy and chemoradiotherapy in esophageal adeno-carcinoma (EAC). J. Clin. Oncol..

[38] Thrift A.P., El-Serag H.B. (2020). Burden of Gastric Cancer. Clin. Gastroenterol. Hepatol..

[39] Fuchs C.S., Shitara K., Di Bartolomeo M., Lonardi S., Al-Batran S., Custem E., Ilson D.H., Alsina M., Chau I., Lacy J. (2019). Ramucirumab with cisplatin and fluoropyrimidine as first-line therapy in patients with metastatic gastric or junctional adenocarci-noma (RAINFALL): A double-blind, randomised, placebo-controlled, phase 3 trial. Lancet. Oncol..

[40] Safran H., Winter K.A., Wigle D.A., DiPetrillo T.A., Haddock M.G., Hong T.S., Leichman L.P., Rajdev L., Resnick M.B., Kachnic L.A. (2020). Trastuzumab with trimodality treatment for esophageal adenocarcinoma with HER2 overexpression: NRG Oncolo-gy/RTOG 1010. J. Clin. Oncol..

